# The Impact of Wettability on Dynamic Fluid Connectivity and Flow Transport Kinetics in Porous Media

**DOI:** 10.1029/2021WR030729

**Published:** 2022-06-03

**Authors:** Rumbidzai A. E. Nhunduru, Amir Jahanbakhsh, Omid Shahrokhi, Krystian L. Wlodarczyk, Susana Garcia, M. Mercedes Maroto‐Valer

**Affiliations:** ^1^ School of Engineering and Physical Sciences Research Centre for Carbon Solutions (RCCS) Heriot‐Watt University Edinburgh UK; ^2^ School of Engineering and Physical Sciences Applied Optics and Photonics (AOP) Group Heriot‐Watt University Edinburgh UK

**Keywords:** wettability, ganglion dynamics, fluid connectivity, fluid flow kinetics, flow resistance, connected pathway flow

## Abstract

Usually, models describing flow and transport for sub‐surface engineering processes at the Darcy‐scale do not take into consideration the effects of pore‐scale flow regimes and fluid connectivity on average flow functions. In this article, we investigate the impact of wettability on pore‐scale flow regimes. We show that fluid connectivity at the pore scale has a significant impact on average flow kinetics and therefore its contribution should not be ignored. Immiscible two‐phase flow simulations were performed in a two‐dimensional model of a Berea sandstone rock for wettability conditions ranging from moderately water‐wet to strongly oil‐wet. The simulation results show that wettability has a strong impact on invading fluid phase connectivity, which subsequently influences flow transport resistance. The effect of invading‐phase connectivity and ganglion dynamics (GD) on two‐phase displacement kinetics was also investigated. It was found that invading phase connectivity decreases away from the neutrally wet (intermediate wet) state. This study provides evidence that GD accelerate fluid flow transport kinetics during immiscible displacement processes. Lastly, the impact of wettability on fluid displacement efficiency and residual saturations was investigated. Maximum displacement efficiency occurred at the neutrally wet state.

## Key Points

- The dominance of ganglion dynamics increases as the wettability shifts away from the neutrally wet state
- Ganglion dynamics accelerate displacement kinetics and reduce resistance to transport of the invading phase
- Fluid displacement efficiency increases as the connected pathway flow regime becomes more dominant

## 1 Introduction

The physical process whereby an immiscible fluid phase replaces a second resident fluid in a porous medium is characteristic of many subsurface operations that include remediation of non‐aqueous phase liquids (NAPLs), enhanced oil recovery (EOR), and carbon capture and storage (CCS) technology (Edery et al., 2018; Singh et al., 2017). Mobilization of residual NAPL and oil blobs and trapping of gas bubbles are critical to such operations (Geistlinger & Mohammadian, 2015).

In CCS applications, the trapping of supercritical carbon dioxide in the interstitial spaces of porous rocks (known as capillary or residual trapping) inhibits plume migration and enhances storage safety and capacity (Al‐Menhali et al., 2016; Krevor et al., 2015). Capillary trapping can contribute up to 40% of the overall CO_2_ trapping in the first 100 years post‐injection (Li et al., 2015), and it is strongly influenced by the wettability of the porous medium (Arif et al., 2017; Basirat et al., 2017; Krevor et al., 2015; Wang et al., 2013). Recently, studies have been conducted using microfluidic devices and numerical simulations to understand the impact of wettability on immiscible displacement (Avendaño et al., 2019; Jahanbakhsh et al., 2021; Wolf et al., 2020). However, how physiochemical interactions between the solid rock minerals and fluid phases affect fluid invasion patterns, displacement efficiency as well as residual saturations in porous media are not fully understood. These are critical parameters in subsurface processes (Zheng & Jang, 2019), where fluids such as CO_2_, brine, and oil have been known to interact and react with rock minerals causing the rocks to deviate from the original strongly water‐wet state by altering the surface chemistry (Kim et al., 2012; Rücker et al., 2019; Seyyedi et al., 2015). This process is known as “wettability alteration” and its significance cannot be ignored. Kim et al. (2012) investigated wettability alteration of silica surfaces by carbonic acid formed by contact reaction of brine and CO_2_ and reported drastic changes of contact angle (CA) from 15° to 80°. Seyyedi et al. (2015) also reported wettability alteration of quartz, mica, and calcite surfaces through contact reaction with carbonated water with a significant change in CA from 144° to 97° occurring for the calcite surfaces.

Pore‐scale properties, such as wettability, and properties pertaining to the physical nature of the porous medium (such as surface roughness, pore size and shape, and pore size distribution) influence Darcy‐scale (macro‐scale) flow functions such as capillary pressure (*P*_c_) and relative permeability (*k*_r_). Although extensive research has been conducted at the core‐scale (centimetre scale) to understand the effects of wettability on *P*_c_ and *k*_r_, correlating these macro‐scale flow functions to pore‐scale properties remains a challenge without performing tedious and extensive lab experiments at micro‐scale. However, successful implementation of CCS and EOR technologies relies heavily on such an understanding.

Multiphase fluid flow phenomena at the micro‐scale can be experimentally investigated using microfluidic devices. Such devices (also called micromodels) typically contain a two‐dimensional (2D) pore network pattern that represents the cross‐section of a natural porous medium. Micromodels allow direct observation of the micro‐scale fluid displacement mechanisms and processes (Grate et al., 2010; Wlodarczyk et al., 2018). Direct numerical simulations (DNS) are also a tool for improving our ability to predict the micro‐scale dynamics of multiphase flow in porous media and bridge the knowledge gap between micro‐ and macro‐scale observations. Advances in computational capabilities in recent years have allowed more research to be conducted using DNS.

Many researchers have sought to gain insight into micro‐scale fluid displacement phenomena through micromodel experiments (Hu et al., 2017b; Jahanbakhsh et al., 2020; Kim et al., 2012; Park et al., 2017; Zhang et al., 2011; Zheng & Jang, 2019) and/or numerical simulations (Azizi et al., 2019; Cao et al., 2016; Ferrari et al., 2015; Hu et al., 2017a; Ryazanov et al., 2014; Wu et al., 2016; B. Zhao et al., 2019).

Micro‐scale fluid flow phenomena involve a combination of two flow regimes: (a) connected pathway flow (CPF) and (b) ganglion dynamics (GD) (Avraam & Payatakes, 1999; Berg et al., 2016; Payatakes et al., 1996). In the CPF regime, the invading phase propagates through the porous medium as a single continuous phase. In the GD regime on the other hand, fluid discontinuity exists in the invading fluid phase and it is transmitted along the porous medium through a series of many irreversible pore‐scale flow phenomena such as mobilization, stranding, breakup, and coalescence (Amili & Yortsos, 2006).

Pore‐scale modeling approaches can be classified as being either quasi‐static or dynamic (Berg et al., 2016). Quasi‐static methods assume that capillary forces alone control interface motion and approximate the fluid displacement process by a series of equilibrium states (local energy minima) at imposed capillary pressures. Such modeling approaches are often insufficient to describe the fluid distributions arising from irreversible displacement events (Armstrong & Berg, 2013; Armstrong et al., 2015; Berg et al., 2016; Helland et al., 2017; Rücker et al., 2015). Standard quasi‐static modeling approaches include pore‐network models which approximate the detailed pore structure by an interconnected network of tubes with idealized geometry and constant cross‐sections. Pore network models use analytic expressions for entry capillary pressures in individual tubes and combine them with invasion percolation algorithms to describe multiphase displacements throughout the network (Helland et al., 2019). As such, one of the major drawbacks associated with quasi‐static approaches is their ability to capture flow only in connected pathway (Berg et al., 2016).

DNS methods, such as the Lattice‐Boltzmann method and Navier‐Stokes approaches, are more intricate and advanced in terms of their ability to accurately capture pore‐scale fluid flow phenomena associated with connected pathway as well as GD. Dynamic DNS models take into account the viscous pressure drops, enabling the capture of the interplay between viscous and capillary forces (Gjennestad et al., 2018). As a result, dynamic modeling methods have gained popularity in recent years as tools for probing pore‐scale fluid dynamics, although their computational cost is much higher in comparison to quasi‐static methods like pore network modeling (PNM) (Berg et al., 2016).

DNS can provide insight into the dynamics of systems, for which a mechanistic description is not readily available (Bureš & Sato, 2021). In this work, we establish the impact of surface wettability on fluid invasion patterns, pore‐scale fluid displacement phenomena, and kinetics through DNS in OpenFOAM v6.

## 2 Materials and Methods

### 2.1 The Volume of Fluid (VoF) Computational Fluid Dynamics Method

The two‐phase incompressible flow solver InterFoam was used to perform 2D simulations in OpenFOAM for immiscible fluid displacement processes in porous media. Fluid flow dynamics are governed by a set of coupled partial differential equations known as the Navier‐Stokes equations. The Navier‐Stokes equations are based on the laws of conservation of mass and momentum (see Equations 1 and 2, respectively), and are solved to determine the fluid velocity (**u**) and pressure (*p*) fields.

(1) $$\frac{\partial \rho }{\partial t}+\nabla \cdot \left(\rho u\right)=0$$

(2) $$\frac{\partial u}{\partial t}+\left(u\cdot \nabla \right)u=-\frac{-1}{\rho }\nabla p+\nu \;\nabla {\;}^{2}+f_{s}$$

where *t* is the time, *ν* the fluid kinematic viscosity, and ***f***_***s***_ is the source term of the momentum due to the surface tension at the interface between the two fluids (*σ*). The ***f***_***s***_ term is expressed as:

(3) $$f_{s}=\sigma \cdot \kappa \cdot n=-\sigma \cdot \nabla \cdot \frac{\nabla \alpha }{\left\|\nabla \alpha \right\|}\cdot \nabla \alpha =0$$

where *α* is a phase indicator function, *κ* the interface curvature, and **n** is the normal vector to the interface (**n** = ∇ *α*) (Hu et al., 2017a). The InterFoam solver uses the VoF phase‐fraction based interface capturing approach to distinguish between two fluids on a computational grid (Hirt & Nichols, 1981). This method has been used to model two phase flow displacement experiments by authors such as Lin et al. (2021) and Subramaniam et al. (2021). With this approach, the system is considered as a medium with a discontinuity in fluid properties at the fluid interface, and a phase indicator function (*α*) is used to track the location of the fluid phases. The indicator *α* symbolizes the concentration or volume fraction of a certain fluid in each cell, and the values of *α* lie between 0 and 1. In the case of two‐phase flow, when a cell is occupied only by fluid 1 then *α* = 1; when a cell is occupied only by fluid 2 then *α* = 0.

(4) $$\alpha \left(x,t\right)\;=\left\{\begin{array}{l} 1\;\text{for}\;x=\text{fluid}\;1\;\text{at}\;\text{time}\;t\\ 0\;\text{for}\;x=\text{fluid}\;2\;\text{at}\;\text{time}\;t \end{array}\right.$$

Intermediate values of *α* represent the interface between the two fluids (Raeini et al., 2015). The evolution of *α* is tracked by an advection‐diffusion equation:

(5) $$\frac{\partial \alpha }{\partial t}+\nabla \cdot \left(\alpha u\right)+\;\nabla \cdot \left(\alpha \cdot \left(1-\alpha \right)\;\kappa \right)\;=0$$

where κ is an artificial compression velocity that supresses numerical diffusion.

DNS takes into account the effect of wettability on fluid flow by imposing boundary constraints on *α* where the fluid–fluid interface and solid surface of the medium coincide (Hu et al., 2017a):

(6) $$\frac{\nabla \alpha }{\left\|\nabla \alpha \right\|\;x\in \text{wall}}=n\;=\;n_{s}\cos\theta +n_{t}\sin\theta \;$$

where **n**_**s**_ is the unit normal to the solid surface and **n**_**t**_ is the unit tangent to the wetting phase.

Equations 1–6 are solved using the so‐called multi‐dimensional limiter for explicit solution (MULES) algorithm. The way in which the MULES algorithm works is that first the pressure and velocity fields are initiated and then the run time loop is started. The loop involves calculating time steps followed by several iterative calculations in which the advection equation is solved and calculation of mixture properties such as density and interfacial tension is done. Lastly, the momentum equation is solved. The solution of the momentum equation (Equation 2) in InterFOAM is performed by constructing a predicted velocity field and then correcting it using the Pressure Implicit with Splitting of Operators correction procedure (Issa, 1986) to time advance the pressure and velocity fields (Deshpande et al., 2012).

### 2.2 Simulation Set‐up

Immiscible two‐phase displacement simulations were performed in a model containing a heterogeneous matrix with a porosity of 32%. The model was a miniature representation of the structure of a Berea sandstone rock sample. The method used to replicate the pore network structure from an X‐ray microtomography image of a Berea sandstone rock was described by Boek and Venturoli (2010) and Wlodarczyk et al. (2019). The microstructure was engineered at Schlumberger Cambridge Research, based on a thin section of a 3D Berea sandstone rock sample imaged by X‐ray microtomography (Boek & Venturoli, 2010). The porous microstructure was discretized on a lattice, and bit‐mapped (0 = pore, 1 = solid grain/obstacle) to generate the matrix (Boek & Venturoli, 2010). The bitmap was then converted to the .stl format which is required for flow simulations in OpenFOAM (Wlodarczyk et al., 2019). This particular microstructure has found use as a benchmark in many complex numerical simulations, such as DNS using OpenFOAM software and lattice‐Boltzmann simulations (Boek & Venturoli, 2010), and in PNM (Mehmani & Tchelepi, 2017) for simulating fluid flow and transport.

**Figure 1:**
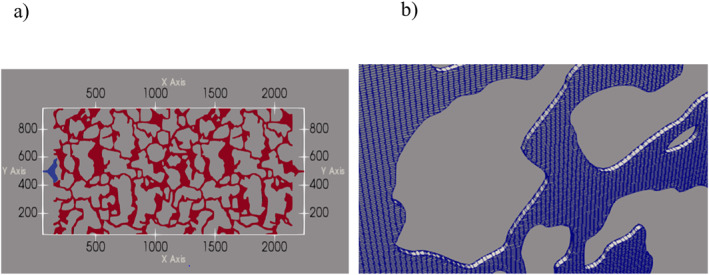
(a) Berea sandstone pore network (model) pattern used in simulations. (b) Snapshot of mesh used to discretize the Berea sandstone model.

The physical dimensions of the model were scaled down 10 times from the original dimensions described by Wlodarczyk et al. (2019) in which the dimensions of the patterned area of the model were 20 mm by 9.1 mm. Using the “scale ‐transformPoints” feature in OpenFoam, the patterned area was scaled down by a factor of 0.1 resulting in the patterned area having dimensions 2 mm (2,000 μm) by 0.91 mm (910 μm) as shown in Figure 1a. The resultant porous structure was not a subset of the model described by Wlodarczyk et al. (2019) but a complete miniature image of the said model. This was done to increase the computational efficiency of simulations in OpenFOAM. When simulations were attempted with the original dimensions of the porous domain, the simulations were heavy (computationally expensive), and convergence issues were encountered. Once the size of the domain was reduced, the problems of convergence were eliminated.

The mesh for the porous matrix (Figure 1b) contained 104,587 cells and 224,302 nodes. It was generated using the “blockMesh” and “snappyHexMesh” utilities in OpenFoam. The mesh had a uniform depth of 5 μm, which was represented by a single cell, therefore there was no flow in the *z*‐direction. The minimum and maximum cell volumes in the mesh were 8.77 and 55.80 μm^3^, respectively. A mesh sensitivity study was conducted in which refining the mesh by a factor of four resulted in no change in residual values (see Figure S1 in Supporting Information S1). It was therefore determined that the chosen mesh refinement was optimum for this study.

For all simulations conducted, similar boundary conditions were applied. The inlet velocity was fixed at 0.3 m/s and a zero‐gradient velocity condition which applies a zero‐gradient condition from the internal field onto the boundary was considered at the outlet. A no‐slip boundary condition was imposed at the walls and a zero‐pressure boundary condition was applied at the outlet. The inlet pressure was then calculated by the InterFOAM solver to satisfy the Navier‐Stokes equations. The value of the contact angle at the fluid–solid interface was the only difference between the simulations performed.

In all the simulations, the Berea sandstone porous structure was initially saturated with decane followed by flooding of water into the system to displace the resident decane phase. The fluid properties of the defending decane and the invading water phase and other flow properties used in the simulations are summarized in Table 1.

**Table 1:** Fluid Properties and Parameters Used in Two‐Phase Flow Simulations

Fluid properties	
Kinematic viscosity of invading water phase (*υ*_inv_)	1E‐06 m^2^/s
Dynamic viscosity of invading water phase (*μ*_inv_)	1.0016 mPa s
Kinematic viscosity of defending fluid (decane) (*υ*_def_)	1.25E‐06 m^2^/s
Dynamic viscosity of defending fluid (decane) (*μ*_def_)	0.920 mPa s
Log *M* (viscosity ratio) or (log *μ*_inv_/*μ*_def_)	0.037
Inlet fluid velocity (*v*_inv_)	0.3 m/s
Reynold's number (Re)	2.117
Water/decane interfacial tension (*σ*)	55 mN/m

The capillary number (Ca) describes the balance between viscous forces and capillary forces and is defined as:

(7) $$\text{Ca}=\frac{\mu_{\text{inv}}\upsilon_{\text{inv}}}{\sigma }$$

where *μ*_inv_ is the dynamic viscosity of invading phase, *v* the invading phase Darcy velocity, and *σ* is the interfacial tension between the invading and defending fluid. Capillary numbers for the simulations performed were in the order log Ca = −3.

The displacement type (capillary fingering, viscous fingering, or stable displacement) for drainage processes is conventionally determined using the viscosity ratio (*M*), capillary number (Ca), and the displacement phase diagram developed by Lenormand et al. (1988). The application of the Ca‐*M* phase diagram has since been extended to imbibition processes (F. Guo & Aryana, 2019). The immiscible fluid displacement processes simulated in this study include both drainage and imbibition, referring to this Ca‐M diagram that was pioneered by Lenormand et al. (1988); we may categorize them under the transition zone between stable displacement, viscous fingering, and capillary fingering invasion patterns.

Binary threshold segmentation was conducted on images extracted from the simulations in the open‐source image analysis software Fiji: Image J (Fiji‐Contributors, 2007), using the “Minimum” thresholding method. This was done to generate residual saturations maps for the simulations performed. These saturation maps allow both a qualitative and quantitative analysis of the pore occupancy during the fluid displacement process at different wettability states.

## 3 Results

### 3.1 Wettability and Pore‐Scale Fluid Displacement Mechanisms

Simulations involving immiscible displacement of decane by water were performed in the Berea sandstone model for both imbibition (water‐wet) and drainage (oil‐wet) processes. The CAs investigated in the study were: 30°, 45°, 90°, 135°, and 150°.

Initially, simulations were performed for CA = 90° (a neutrally wet state) as well as values 45° on either side of the neutrally wet state, that is, CA = 45° (a moderately water wet state) and CA = 135° (a moderately oil wet state); however, after observing and analyzing kinetic trends in these chosen cases, it was decided to conduct additional simulations for CA values of 60° on either side of the neutrally wet state, that is, CA = 30°(a strongly water wet state) and CA = 150°(a strongly oil wet state) were performed. Therefore, in this article, the 90° and 90° ±45° simulation cases are described first followed by the 90° ± 60° simulation cases.

**Figure 2:**
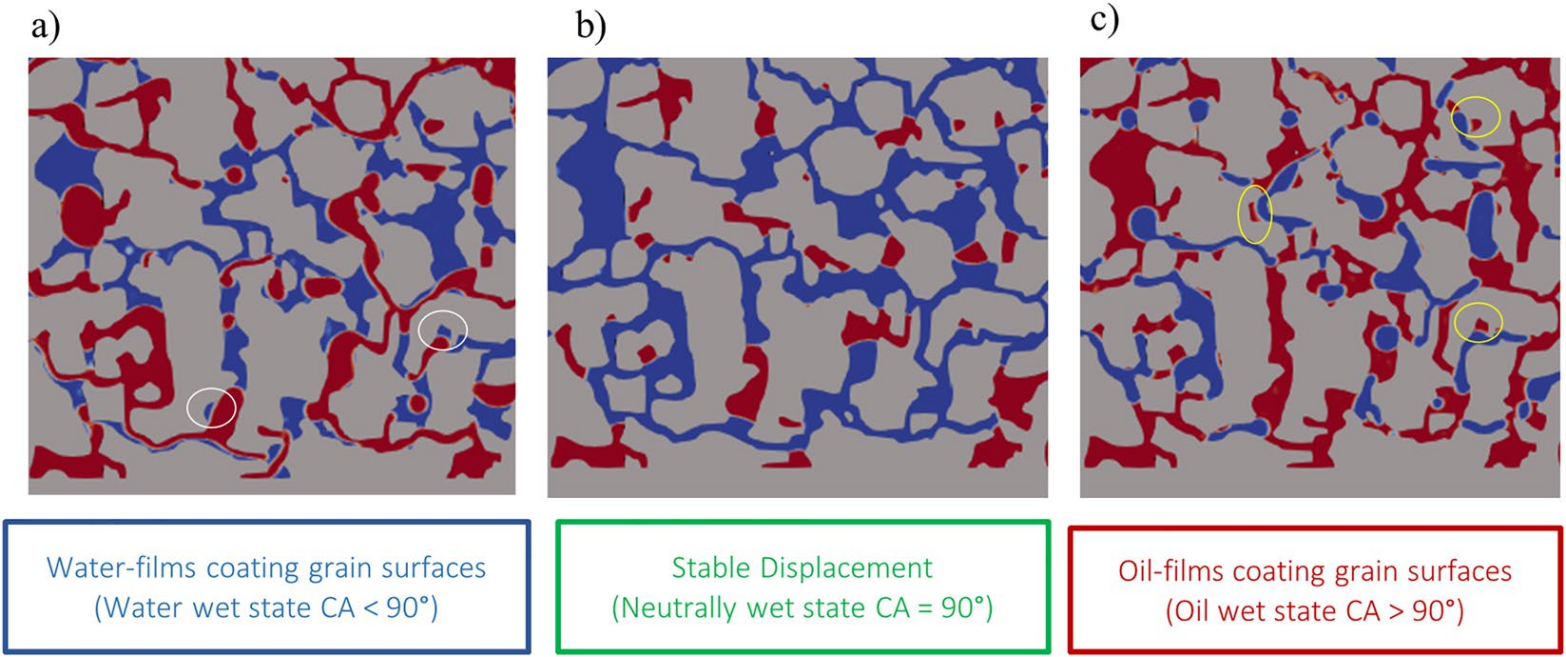
Fluid displacement patterns observed in the Berea sandstone model. (a) Corner flow—water films coating the grain surfaces under water‐wet conditions (imbibition). (b) Stable, piston‐like displacement (under neutrally wet‐conditions). (c) Films of oil coating the grain surfaces under oil‐wet conditions. White circles show crevices occupied by the water phase (blue) and yellow circles show crevices occupied by the decane phase (red).

During imbibition (see Figure 2a), when the porous microstructure had a high affinity for water, it was observed that the invading water phase (in the form of water films) preferentially coated (adhered to) the walls of the grains as it advanced forward into the porous medium. Because the affinity of the medium to invading water phase was high, the water was able to displace the defending decane phase from the small corners and crevices (see white circles in Figure 2a) during its advance (corner‐flow). Corner flow has also been observed for imbibition by several authors including X. Guo et al. (2020) and Bakhshian et al. (2021).

Corner flow impedes piston‐like advance. This results in incomplete fluid displacement and promotes snap‐off events (X. Guo et al., 2020). In imbibition, the defending non‐wetting phase is trapped in the central region of large pore bodies due to the wetting fluid preferentially coating the surface of the matrix.

In the neutrally wet 90° case (see Figure 2b), the porous matrix had no preferred affinity for either the invading water phase or the defending decane phase. As such, no preferential coating of the matrix by either fluid occurred and the invading water front propagated in a compact and stable manner as reported by Hu et al. (2018) for similar capillary number (log Ca = −3) and contact angle. The stable front results in efficient (complete) displacement.

Under the oil wet condition (see Figure 2c), the porous medium had a high affinity for the defending decane phase. As a result, thin films of the decane phase coated the grain surfaces and occupied crevices of the porous medium (see yellow circles in Figure 2c), while the non‐wetting water phase propagated within the central regions of the narrow throats. Zacharoudiou et al. (2020) also reported that the presence of the surrounding wetting phase films causes snap‐off and disconnection of the invading non‐wetting phase in the narrow‐constricted pore throats.

### 3.2 The Effect of Wettability on Advancing Front Speed and Fluid Invasion Patterns

**Figure 3:**
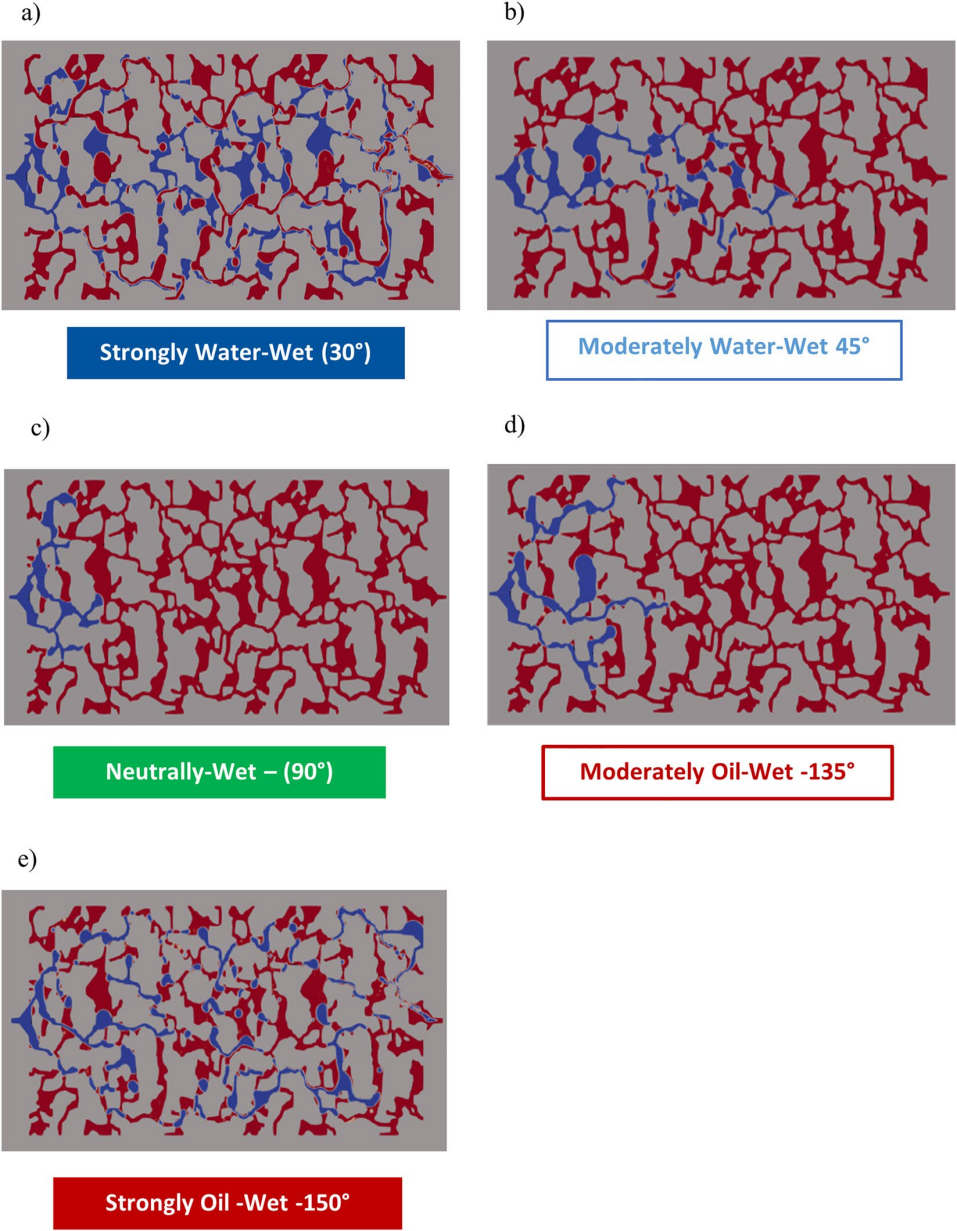
Fluid distribution patterns at *t* = 0.009 s for (a) CA = 30°, (b) CA = 45°, (c) CA = 90°, (d) CA = 135°, and (e) CA = 150°. In all snapshot images from simulations shown, the water phase is shown in blue, while the decane phase is shown in red, and the direction of fluid flow is from left to right.

Different fluid displacement mechanisms were observed for the water wet, neutrally wet, and oil‐wet cases (Figure 3)

#### 3.2.1 Comparing Flow Transport Behavior for CA Values 90° and 90° ± 45°

When the porous matrix was moderately water‐wet (CA = 45°), preferential channeling occurred, with the invading waterfront propagating in the form of a “long finger” toward the outlet (see Figure 3b). This preferential channeling of flow resulted in large sections of the defending decane phase (red color) being by‐passed by the invading water phase (blue color). For CA = 90° and 135° (see Figures 3c and 3d, respectively), no preferential flow channeling occurred, and a more lateral and uniform spread of the invading fluid was instead observed.

The primary difference between fluid invasion patterns in the CA = 90° and 135° cases was the connectivity of the invading water phase. For CA = 90° the invading water phase did not disintegrate, as shown in Figure 3c, while for CA = 135° the invading water phase disintegrated during the fluid invasion process, as shown in Figure 3d (also see the supplementary videos for these simulation cases—Movies S3 and S4).

Less water invasion in the CA = 90° simulation case (Figure 3c) in comparison to the CA = 45° and CA = 135° simulation cases (Figures 3b and 3d) indicates that the lateral invasion and high connectivity of the invading water phase significantly slowed down the propagating front. This observation provides strong evidence that wettability affects flow transport kinetics. Gharbi and Blunt (2012) also observed that both connectivity and the wetting state of the porous media affect the fluid flow transport resistance. J. Zhao et al. (2018) reported similar observations and attributed this phenomenon to wettability‐related micro‐scale fluid distributions. In this study, we visualize and quantify the effects of this phenomenon at the pore‐scale.

**Figure 4:**
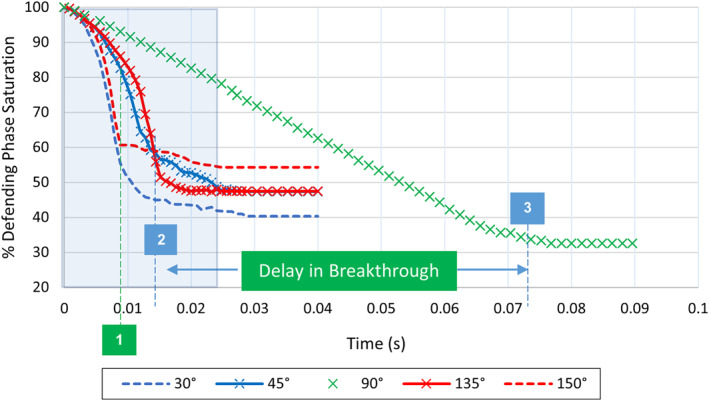
Decane desaturation curves for CA = 30°, 45°, 90°, 135°, and 150° in Berea sandstone model. The time point labeled 1 (curve inflection point) refer to the times at which fluid breakthrough occurred in the 30° and 150° cases, point 2 refers to breakthrough time in 45° and 135° cases, and point 3 refers to breakthrough time in 90° case. Highlighted blue region is magnified and presented in detail in Figure 5.

In the neutrally wet CA = 90° simulation case, the invading front advanced the least distance after 0.009 s in comparison to the other wet cases (see Figure 3), and the so‐called breakthrough, that is (when the invading fluid reached the outlet) occurred much later under this wetting condition in comparison to oil and water‐wet conditions. Breakthrough occurred at around 0.0126 s in the water‐wet state (CA = 45°) and at around 0.0152 s in the oil‐wet state (CA = 135°) (Figure 4).

The delay in breakthrough, however, was much more pronounced in the CA = 90° case, where breakthrough occurred after 0.0720 s (Figure 4). There was an average delay of 0.0581 s in breakthrough time between the CA = 90° case and the cases where CA = 45° and 135°. The propagating front in the CA = 90°, neutrally wet simulation was roughly five times slower in reaching the outlet than that in the CA = 45° and 135° simulations, as seen in Figure 4.

This lag/delay in breakthrough time is attributable to the change in pore‐scale flow regime that accompanies a change in wettability is represented by the difference in *x*‐axis values of the inflection points (points at which a curve changes direction or gradient) of the CA = 45°, 90°, and 135° desaturation curves (points 2 and 3 shown by the blue, vertical dashed lines) in Figure 4. Farad et al. (2016) also reported that breakthrough time is influenced by the wettability of the porous medium. Fluid disintegration occurred in the non‐neutrally wet CA = 45° and 135° simulation cases. The point at which fluid disintegration commenced is shown by “point 1” in Figure 4.

Although different fluid invasion patterns were observed in the simulations for CA = 45° and 135° (Figures 2b and 2d), it is interesting to note that the similar trends are observed in the desaturation curves for these two cases during the rapid desaturation phase as shown in Figure 4. This implies that for these two different wettability conditions 45° on either side of the neutrally wet state (described as CA = 90° ± 45°), the rate of change of defending phase followed a similar trend with slight differences in breakthrough time and residual saturations (curve tail end values) observed. To the best of our knowledge, such kinetic behavior has not yet been reported in the literature.

#### 3.2.2 Comparing Flow Transport Behavior for CA Values 90° and 90° ± 60°

To further investigate this kinetic phenomenon, water flooding simulations were also performed for CA values of 60° on either side of the neutrally wet state (i.e., for CA = 30° and 150°). As was observed for the CA = 45° and CA = 135° cases, similar desaturation trends were also observed for the CA = 30° and 150° cases, as can be seen in Figure 4.

Like in the non‐neutrally wet CA = 45° and 135° simulation cases described above, the invading water phase in the CA = 30° and CA = 150° simulations was highly disconnected during the fluid displacement process (Figures 3a and 3e). Interestingly, the fluid displacement rates in the simulations performed for CA = 30° and 150° were greater than the rates observed in the simulations performed for CA = 45° and 135°, as shown in Figures 3a and 3e.

In Figure 3, after 0.009 s the invading water phase had reached the outlet in the CA = 30° and 150° simulations (Figures 3a and 3e) and in the CA = 45° and 135° simulations (Figures 3b and 3d) it had advanced roughly half the length of the Berea sandstone porous model. The slowest fluid advance occurred in the CA = 90° simulation case (Figure 3c). This is also seen in the desaturation plot for the simulations (Figure 4) in which the CA = 30° and 150° simulation cases (red and blue dotted curves in Figure 4) have the steepest slope followed by the CA = 45° and 135° cases (red and blue solid line curves with crosses in Figure 4). The CA = 90° simulation case has the least steep slope in Figure 4, indicating that the rate of fluid displacement (front speed) was slowest in this simulation case.

### 3.3 The Effect of Ganglion Dynamics Onset on Desaturation Kinetics

Two pore‐scale flow regimes were observed and identified: (a) the CPF regime in which there is no discontinuity of the invading fluid phase and (b) the GD flow regime in which there is discontinuity of the invading fluid phase. These flow regimes were also reported by Payatakes et al. (1996) in micromodel studies and later by authors including Berg et al. (2016), Reynolds et al. (2017), and Rücker et al. (2015, 2019) in studies using rock cores and X‐ray computed microtomography with ultrafast synchrotron sources.

In this study, the invading water phase was continuous throughout the entire structure for the neutrally wet, CA = 90° state with phase discontinuity being observed in all other wetting states investigated due to the occurrence of events such as breakup, coalescence, stranding, and mobilization (GD)—see Movies [Link], [Link], [Link], [Link], [Link].

**Figure 5:**
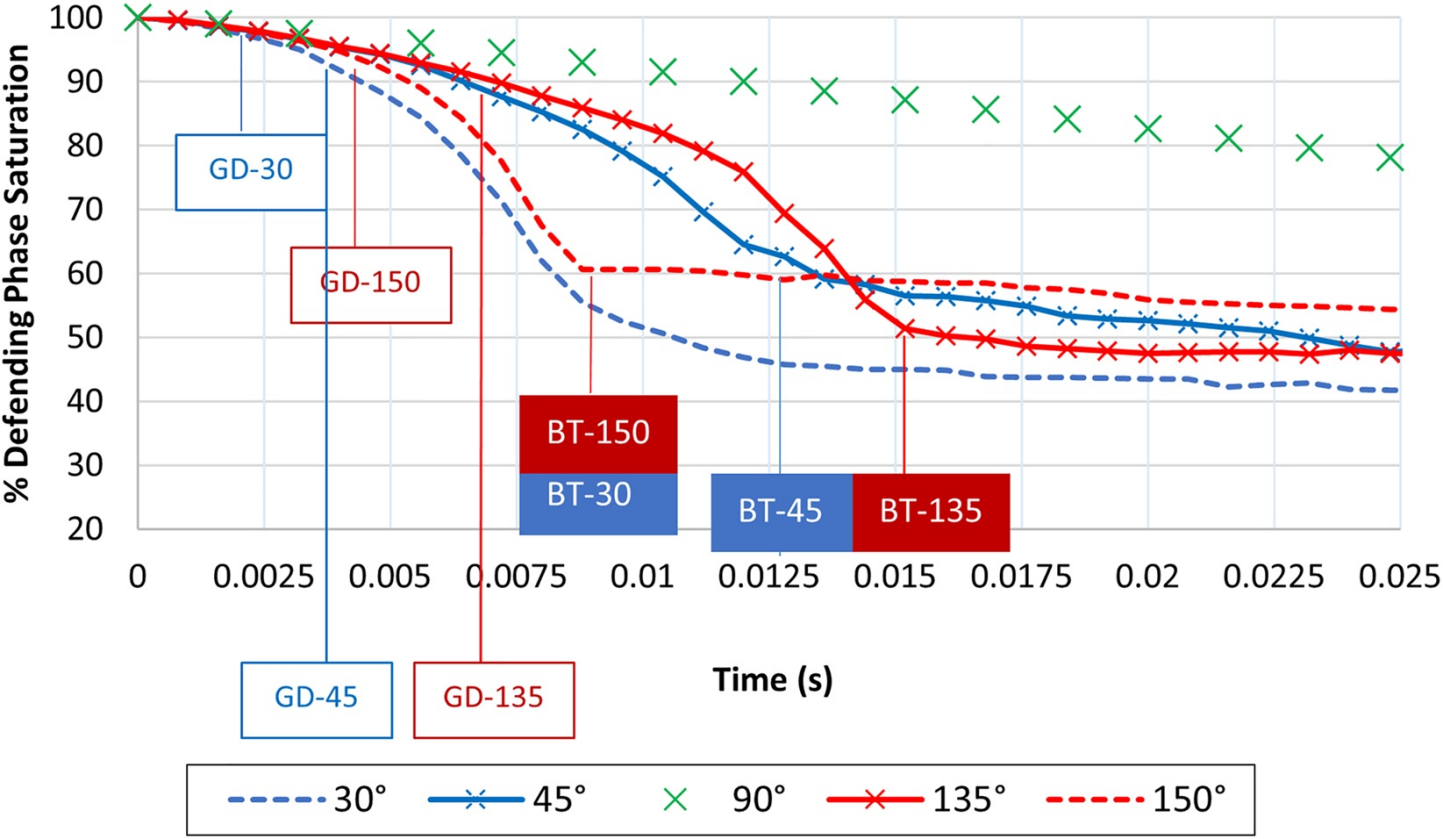
Magnification of first 0.025 s of simulations (section highlighted in blue in Figure 4) for CA = 30°, 45°, 90°, 135°, and 150° desaturation curves in the Berea sandstone model. Solid colored blocks refer to BT (breakthrough times) and white filled blocks refer to GD (ganglion dynamics onset times). Red color denotes oil wet cases while blue denotes water‐wet cases.

Comparing dominance of flow regimes with time in the simulations, we find that a transition from the CPF to the ganglion flow regime occurs with the dominance of GD increasing as the medium becomes more oil‐ or water‐wet. We analyze the effect of this transition on desaturation kinetics. It is observed that the onset of GD occurs much earlier as the medium becomes more water‐wet or oil‐wet. To show this the first 0.025 s of the simulations (area highlighted in blue in Figure 4) is plotted in Figure 5, which is a magnified version of Figure 4.

In the initial stages of all the simulations, the invading water phase was continuous (entirely connected), and the CPF mechanism dominated. In all simulation cases other than the neutrally wet CA = 90° case, after a certain time, the onset of GD events such as break up, mobilization coalescence and stranding of ganglia of the invading phase occurred (also known as GD onset).

GD onset can be determined using the time‐saturation data in Figures 4 and 5 in which the onset of GD events leads to an abrupt increase in the rate of fluid displacement. This is evidenced by inflection points in the saturation data whereby the gradient of the saturation curves increases. The onset of GD was also determined by visual inspection of the simulations (see Movies S1, S2, S4, and S5) which coincides with the inflection points observed in the saturation data curves. Once the first GD event occurs, it triggers a sudden “avalanche” of events. GD rapidly accelerate the desaturation process as such, there is a change in the gradient of the desaturation curve (an inflection point). As the frequency of GD increases within a given system, the steepness of the desaturation curve also increases.

While the saturation remained nearly constant with slight decreases in value observed after breakthrough in the none neutrally wet scenarios (i.e., CA = 30°, 45°, 135°, and 150° cluster disconnection, reconnection, and mobilization events continued to occur in these cases) the systems remained highly dynamic after breakthrough (see Movies S1, S2, S4, and S5).

#### 3.3.1 GD Onset Comparison for CA Values 90° and 90° ± 45°

Considering the CA = 45° and 135° (CA = 90° ± 45°) simulation cases, in the region in which CPF dominated (region to the left of the green line labeled “1” in Figure 4), the defending phase saturation decreased at a slower rate. However, the onset of GD rapidly accelerated the rate of change of defending phase saturation resulting in a sharp change in curve gradient at the inflection point shown by the green line labeled “1” in Figure 4. Disintegration of the invading water phase began to occur after 0.0034 s in the water‐wet CA = 45° case and after 0.0068 s in the oil‐wet CA = 135° case (labeled GD‐45 and GD‐135, respectively, in Figure 5).

Comparing the snapshot images for the CA = 45° and 135° simulation cases at a simulation time of 0.0009 s in Figure 2, the invading fluid phase is more fragmented in the water‐wet CA = 45° case than in the oil‐wet CA = 135° case as the onset of GD had occurred much earlier and more GD events had occurred in the CA = 45° case at the said time.

Because the onset of GD events occurred much earlier in the CA = 45° simulation case, the fluid reached the outlet (occurrence of breakthrough) faster (at *t* = 0.0126 s, represented as BT‐45 in Figure 5) than in the CA = 135° case (at *t* = 0.0150 s, represented as BT‐135 in Figure 5). Following breakthrough, the saturation assumes a nearly constant tail‐end value which slowly decreases with time as the fluid ganglia approach dynamic equilibrium and stability. In the CA = 90° case the invading phase remained entirely connected until it reached the outlet—see Movies [Link], [Link], [Link], [Link], [Link]—as the gradient of the curve did not change until the breakthrough point shown by the blue line labeled “3” in Figure 4. Our findings support those by Berg et al. (2016) that GD not only contribute toward the flux of fluids but also impact the time evolution of the pore‐scale fluid distributions, and the configuration of the connected phases (Berg et al., 2016).

#### 3.3.2 GD Onset Comparison for CA Values 90° and 90° ± 60°

The onset of GD was found to occur much earlier in the strongly water‐wet and oil‐wet CA = 30° and 150° simulation cases in comparison to the CA = 45° and 135° cases. As such, the low gradient region in which CPF dominates and the rate of saturation change is slow, prevailed for less time on the curves for the CA = 30° and 150° simulation cases in comparison to CA = 45° and 135° cases (Figure 5).

Breakthrough also occurred much earlier in these cases (at *t* = 0.0086 s and at 0.0088 s in the CA = 30° and 150° cases, respectively)—shown in Figure 5 as BT‐30 and BT‐150. While the onset of GD occurred at almost the same time in these two cases, there is a significant difference in tail end saturations (57% in the CA = 150° case and 44% in the CA = 30° case). Breakthrough times were very similar for CA = 45° and 135°, as well as for CA = 30° and 150°. A summary of the GD onset and breakthrough times is given in Table 2.

**Table 2:** GD Onset and Breakthrough Times for CA of 30°, 45°, 90°, 135°, and 150°

CA	GD onset time (s)	Breakthrough time (s)
30°	0.0014	0.0086
45°	0.0034	0.0126
90°		0.072
135°	0.0068	0.0150
150°	0.0042	0.0088

This behavior can be attributed to the connectivity of the invading water phase in the two cases. At any given time in the network of pores, where GD occurs, the invading fluid phase is not necessarily connected. As reported by Reynolds et al. (2017), flow occurs along pathways that periodically disconnect and reconnect like cars controlled by traffic lights. This type of behavior is consistent with an energy balance, where some of the energy of the injected fluids is sporadically converted to create new interfaces (Reynolds et al., 2017).

In the strongly water‐wet CA = 30° and strongly oil‐wet CA = 150° cases, the invading water‐phase was highly discontinuous and existed as several individual ganglia throughout the medium that periodically connected and disconnected during the displacement process. A relationship exists, between the number of connected flow pathways and the displacement efficiency with the displacement efficiency being higher where more connected flow pathways of the invading fluid exist.

### 3.4 Wettability and Temporal Evolution of Defending Phase Ganglia

#### 3.4.1 Ganglia Quantification

**Figure 6:**
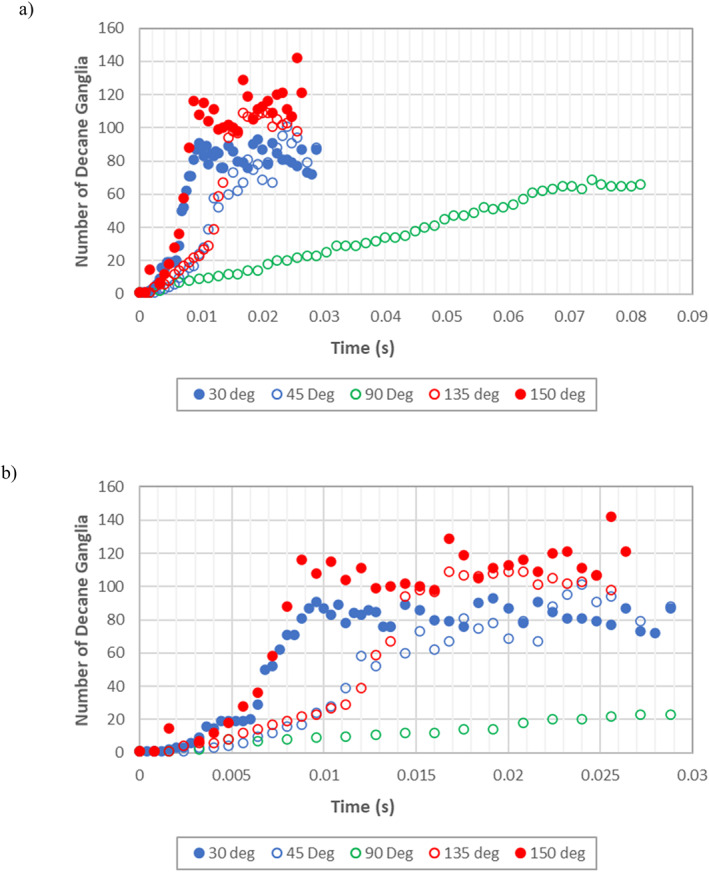
(a) Time evolution of the defending phase ganglion count for CA = 30°, 45°, 90°, 135°, and 150° simulation cases in Berea sandstone model (0–0.09 s). (b) Magnification of first 0.03 s of defending phase ganglion count for CA = 30°, 45°, 90°, 135°, and 150° simulation cases in Berea sandstone model.

To fully understand the effects of wettability on dynamic fluid connectivity and the changes in ganglion behavior over time, ganglia of the defending decane phase and their areas were quantified using the image processing and analysis software FIJI‐Image J. A code was written in Python scripting language to instruct the software to count the number of individual objects (ganglia) in a binary image and measure the area and perimeter of each individual ganglion—see Figure S2 in Supporting Information S1. The number of ganglia of the defending decane phase present in the porous medium as the fluid invasion process progressed were quantified for all the simulation cases investigated (Figure 6).

In Figure 6a for all the simulation cases investigated, the general trend is that the number of ganglia of the defending decane phase present in the porous system increased with time during the displacement process. This indicates that in all instances, the defending phase gradually became more and more disconnected over time as the water phase invaded the pore spaces. Following breakthrough, the rate at which the defending phase becomes disconnected slows down and the curves reach a plateau. Examining the fluid displacement processes in more detail, in Figures 6a and 6b the slowest increase in the number of decane ganglia occurred in the neutrally wet, CA = 90° case. Due to dominance of the CPF regime in the 90° case, the displacement process and subsequent disconnection of the defending phase occurs at a very slow rate in comparison to the other cases. As only the CPF regime dominated in this case the rate at which the defending phase became disconnected remained almost constant during the displacement process, that is, the slope of the 90° case remained until constant breakthrough was achieved (Figure 6a). Once breakthrough occurred, dynamic stability was achieved in the system. No further disconnection of the decane phase occurred and the number of decane ganglia present in the system remained constant.

Initially, the desaturation rate in the non‐neutrally wet cases (CA = 30°, 45°, 135°, and 150°) was the same as in the neutrally wet 90° case due to dominance of the CPF regime at the start of all simulations. As time progressed (Figure 6b), the rate of disconnection of the defending phase (slope of the curves) increased due to the onset of GD in the non‐neutrally wet cases. Step wise increments in the rate of change of ganglia are observed in the CA = 30°, 45°, 135°, and 150° cases (Figure 6b). This is because the frequency of occurrence of GD events increased as the fluid displacement process progressed. The highly oil‐wet (CA = 150°) case and the highly water wet 30° case disconnected at a faster rate than the less oil‐wet (CA = 135°) case and the less water‐wet (CA = 45°) case.

One key difference between the neutrally wet case (CA = 90°) and the non‐neutrally wet cases (CA ≠ 90°) in Figure 6 is that following breakthrough in the non‐neutrally wet cases fluctuations in the number of defending phase ganglia is observed while in the neutrally wet 90° case the number of ganglia remained constant. This is because following breakthrough in the non‐neutrally wet CA = 30°, 45°, 135°, and 150° cases, the system remained highly dynamic. Ganglion break‐up, mobilization, merge, and coalescence events continued to occur post breakthrough for both phases (see Movies [Link], [Link], [Link], [Link], [Link]). While the system exhibited post‐breakthrough dynamic behavior in these cases, the fluid saturations remained at a near constant value as observed in Figure 4. This is because the invading fluid flow paths would have been fully developed when breakthrough is achieved. Fluid that is injected after breakthrough has occurred follows the fluid displacement path developed prior to breakthrough and very little additional displacement of the defending fluid occurs after breakthrough. The least number of ganglia following breakthrough is observed in the 90° case in Figure 6a, that is, the defending phase is least disintegrated in the 90° case in comparison to other cases and this can be attributed to dominance of the CPF regime. From Figure 6, it can be deduced that increasing the hydrophobicity or hydrophilicity of a system decreases the connectivity of the defending phase due to increased occurrence of GD events.

#### 3.4.2 Time Dependent Ganglion Size Distribution and Residual Saturation Mapping

**Figure 7:**
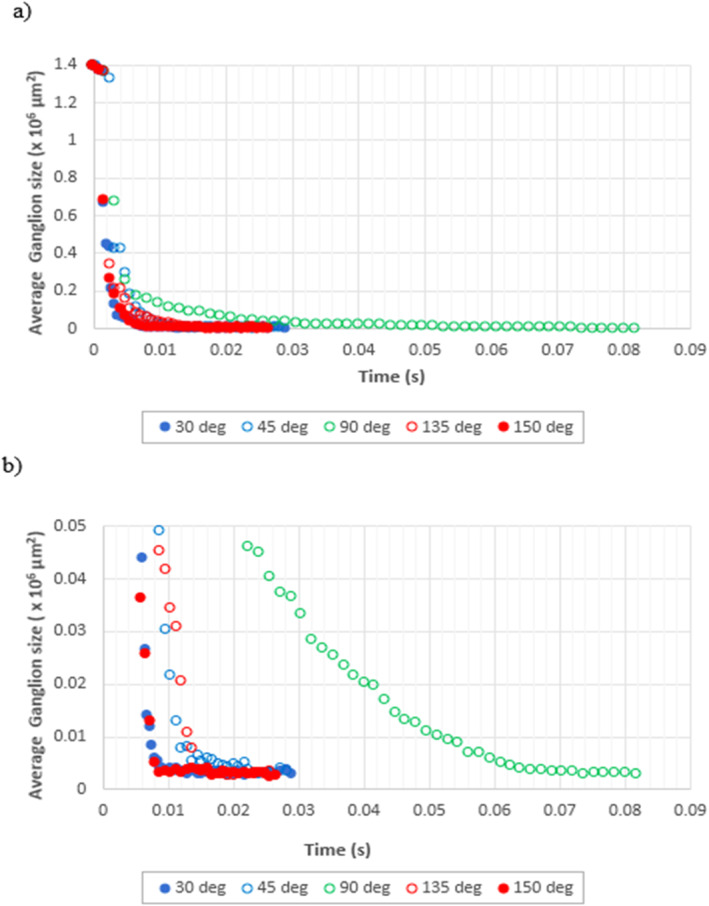
Time evolution of the average size of ganglia of defending decane phase present in the Berea sandstone structure for CA 30°, 45°, 90°, 135°, and 150° for (a) time range 0–0.09 s and (b) magnification of (a).

To gain deeper insight into the effects of wettability on the temporal evolution of the defending fluid ganglia, the change in average size of the ganglia observed in the porous system as the fluid displacement process progressed was tracked for CA of 30°, 45°, 90°, 135°, and 150° (Figure 7).

For all the simulation cases investigated, the average size of defending phase ganglia present in the system decreased with time as the advancing water phase invaded pore spaces subsequently displacing and disintegrating the defending phase into smaller ganglia (Figure 7). As the Berea sandstone microstructure was initially fully saturated with the decane, the initial size of the decane ganglia was the same (1.39 mm^2^) for all cases investigated. Following breakthrough, the average size of the defending phase ganglia tails off and assumes a constant or near constant value once breakthrough has been achieved (Figure 7). The average residual ganglion size ranged from 2,963 to 3,345 μm^2^ as shown in Table 3 in which a summary of the residual ganglion characteristics for all the wetting conditions investigated is presented.

**Table 3:** Residual Ganglion Characteristics for CA of 30°, 45°, 90°, 135°, and 150°

CA	Number of ganglia	Total ganglion area (μm^2^)	Average ganglion size (μm^2^)	Average ganglion perimeter (μm)
30°	87	257,770	2,963	276
45°	85	312,050	3,671	335
90°	65	203,275	3,127	272
135°	98	327,808	3,345	311
150°	113	338,879	2,999	285

While all simulations eventually reach an average residual ganglion size of around 0.0033 × 10^6^ μm^2^ (3,300 µm^2^), in Figures 7a and 7b, the average ganglion size decreased at the slowest rate in the neutrally wet, 90° simulation case in which the CPF regime dominated. In this case the residual ganglion value was achieved at 0.068 s. The rate of decrease of the average ganglion size is about five times much faster in the 45° and 135° cases with the residual value being achieved at around 0.0135 s in which a transition from the CPF to the GD regime occurred. The rate of decrease of the average ganglion size was even fastest in the 30° and 150° cases in which early onset of the GD regime occurred with the residual ganglion size being achieved after about 0.0085 s (Figures 7a and 7b).

**Figure 8:**
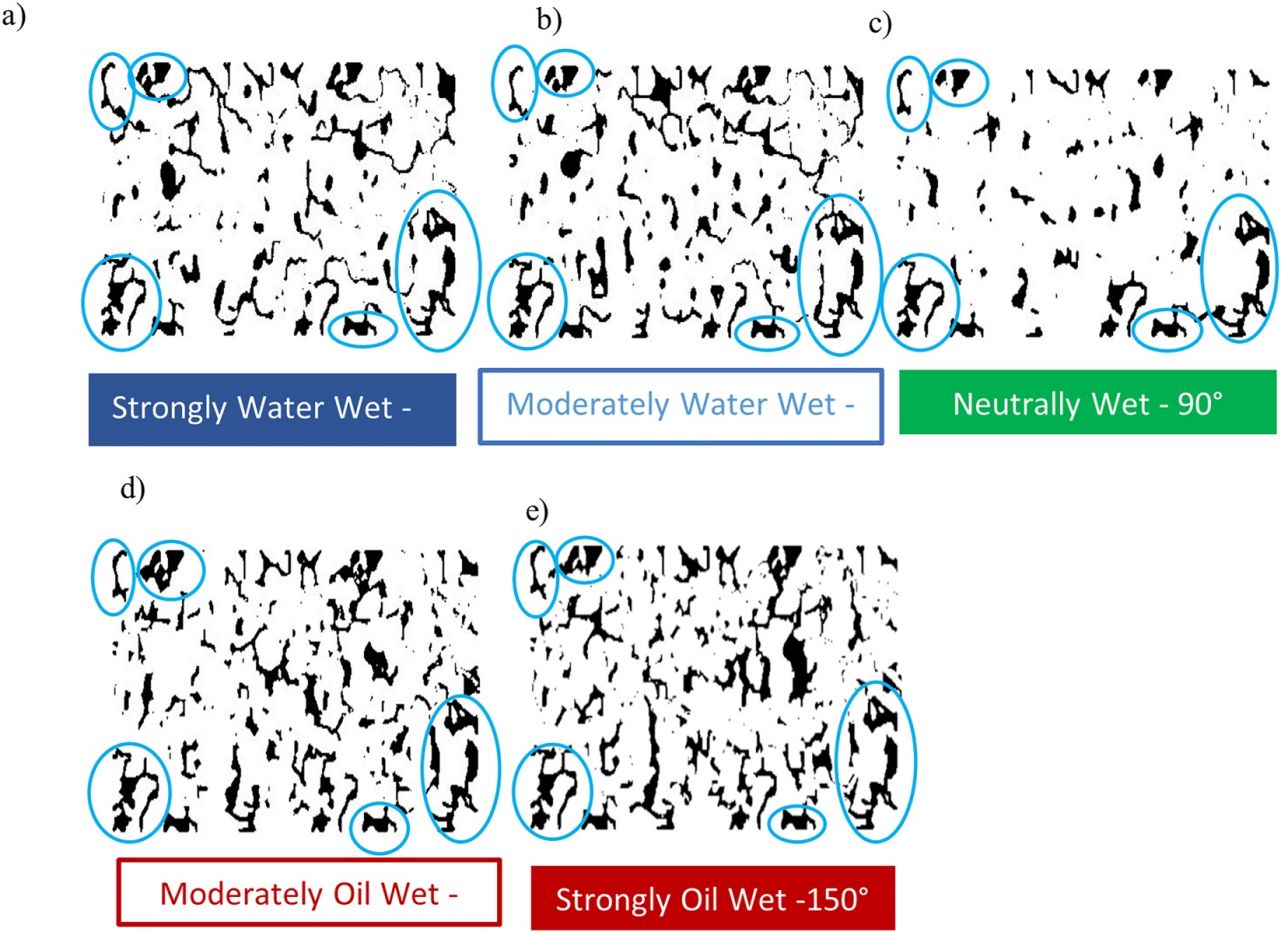
Residual saturation maps for (a) CA = 30°, (b) CA = 45°, (c) CA = 90°, (d) CA = 135°, and (e) CA = 150° in Berea sandstone model. The black color denotes pore regions occupied by residual decane and blue circles show examples of dead‐end type pores in the Berea sandstone structure studied.

Residual saturation maps were also generated for all the simulation cases (Figure 8). Here fluid displacement efficiency is seen to be highest in the neutrally wet state (CA = 90°) with the defending phase having been displaced from most pores within the structure. Most of the trapped clusters of the defending phase under such wetting conditions are seen to occupy individual pores in Figure 8c. The residual (trapped) saturation in this case was 33%. Trapping was more pronounced in the water‐wet and oil‐wet cases (Figures 8a, 8b, 8d, and 8e). Enhanced break‐up of the invading water phase due to GD events resulted in less efficient (incomplete) displacement of the resident decane phase and higher residual saturation under such conditions.

In the water‐wet CA = 30° and 45° and oil‐wet CA = 135° and 150° cases, shown in Figures 8a, 8b, 8d, and 8e respectively, large clusters of the defending decane phase, spanning over multiple pores, were observed. In contrast, single pore occupancy was observed in the neutrally wet case. Movement of these large clusters through the pore space occurred through a series of breakup and coalescence events. In Figure 8 it can be observed that the number and size of isolated, trapped ganglia increases as the medium becomes more water‐ or oil‐wet with more ganglia observed in the oil‐wet cases. It should be noted that while large ganglia spanning multiple pores occurred in the non‐neutrally wet scenarios (Figure 8), these ganglia did not result in notable differences in the average residual ganglion size. This is because most of the residual ganglia were small (on average about 3,300 µm^2^) in all the cases investigated while the larger ganglia (as large as up to 29,000 µm^2^ in size) were few (see residual ganglion size distribution plots in Figures S3–S7 of Supporting Information S1).

**Figure 9:**
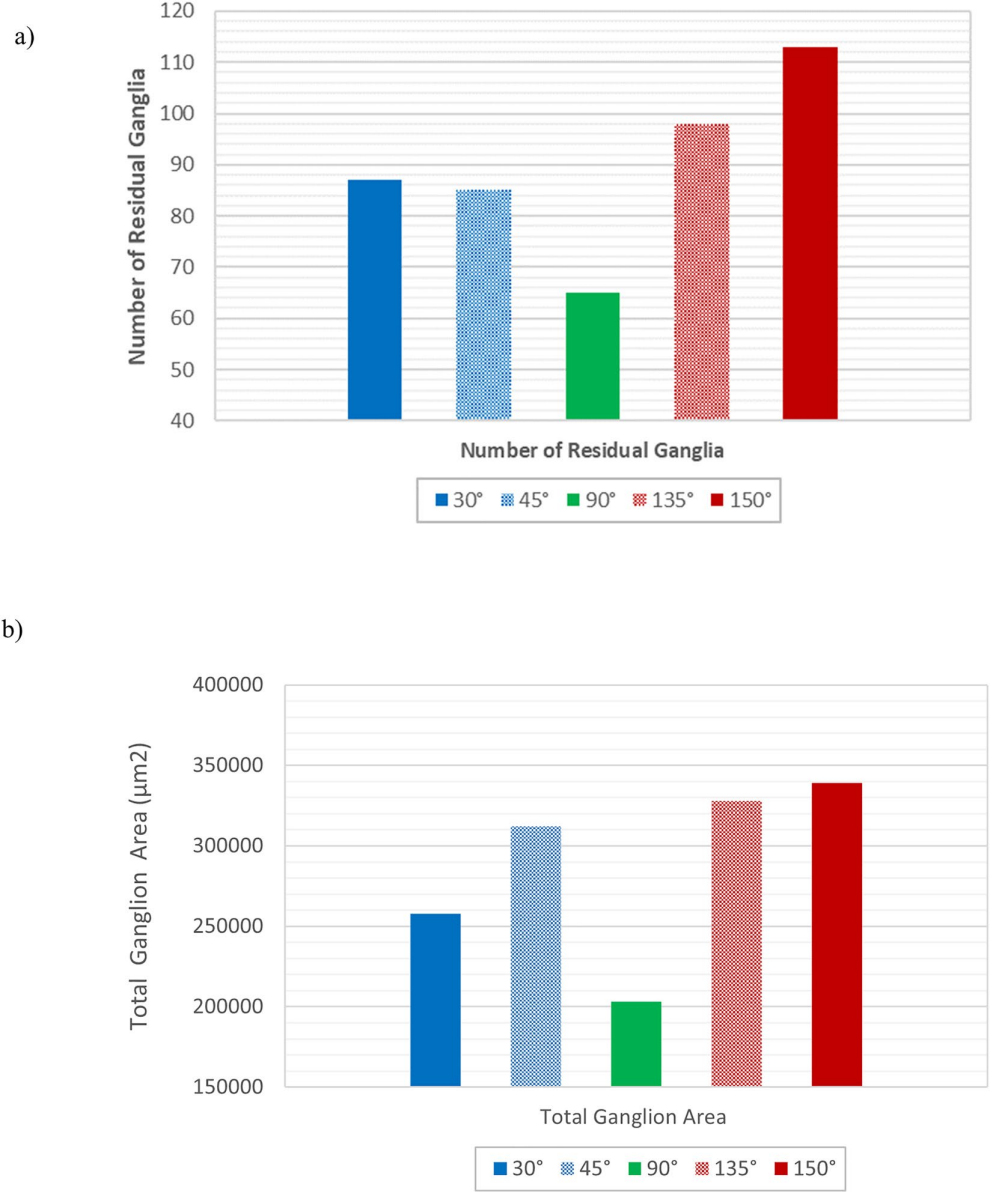
(a) Number of residual ganglia and (b) total residual ganglion area in Berea sandstone microstructure for CA = 45°, 90°, 135°, and 150°.

While large ganglia were few in the non‐neutrally wet 30°, 45°, 135°, and 150° simulation cases, their contribution to the total residual ganglion area was significant with notable differences in total ganglion area between the neutrally wet, 90° case in which single pore occupancy occurred and in the non‐neutrally wet cases (Figures 8 and 9b).

In Figures 8 and 9b the total residual ganglion areas in the oil‐wet cases (135° and 150°) in which GD dominated and multiple pore occupancy occurred were 1.61 and 1.67 times larger than neutrally wet 90° in which the CPF regime dominated, and single pore occupancy occurred. While still significantly larger than in the neutrally wet 90° simulation case, the total residual ganglion areas in the water‐wet cases (30° and 45°) were lower than in the oil‐wet cases. Higher residual saturations in the oil‐wet cases can be attributed to the medium's high affinity for the defending decane phase in these cases and incomplete fluid displacement due to the GD flow regime.

Despite significant differences in saturations, the images in Figure 8 suggest dominance of structural effects on capillary trapping as certain pores (circled in blue in Figure 8) retain trapped defending phase regardless of the wettability of the system. Most of these pores were identified as being dead‐end type pores at the boundaries of the Berea sandstone structure studied. The presence of these dead‐end type pores (a boundary effect) was found to enhance the irreducible saturation of the defending decane phase in the porous medium, as the fluid contained in such pores is trapped permanently and cannot be displaced from the pocket. This is undesirable in operations, such as CO_2_ storage and enhanced oil recovery, where the primary objective is to displace the resident fluid (either oil or brine) from the rock pores.

**Figure 10:**
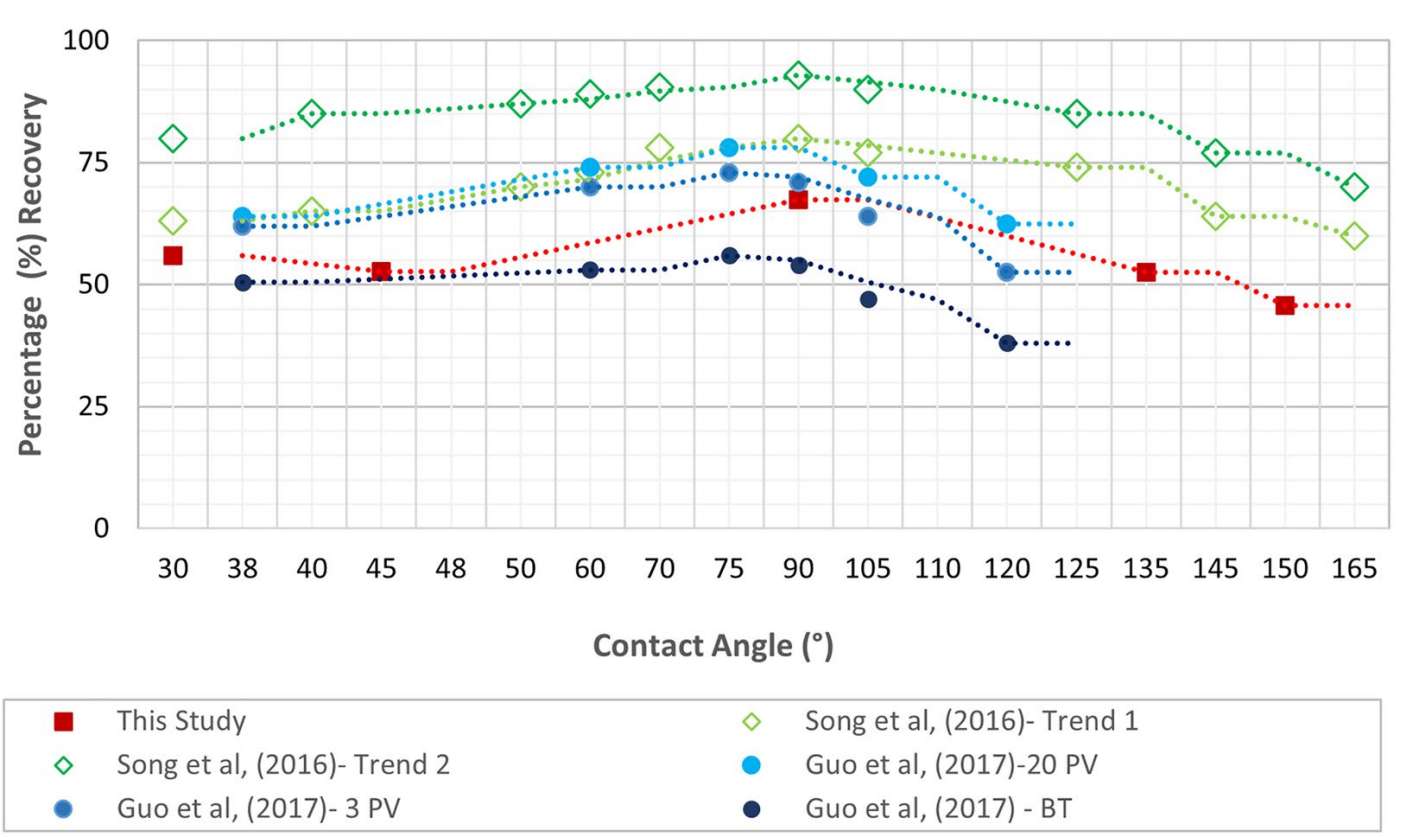
Percentage recovery versus CA for this study compared with Song et al. (2016) and H. Guo et al. (2017).

Figure 10 depicts the variation of percentage recovery of the defending phase with CA. The trend observed here is in strong agreement with those previously observed by other authors (Alhammadi et al., 2017; H. Guo et al., 2017; Song et al., 2016; X. Zhao et al., 2010) in which maximum recovery occurs close to the neutrally‐wet state conditions and decreases away. While our data follow a similar trend to that of Song et al. (2016), residual saturations in this study are slightly higher because of the many dead end pores at the boundaries of the simulation model.

## 4 Conclusions

In this work, the effect of wettability on desaturation kinetics and pore‐scale capillary trapping mechanisms are investigated through direct numerical simulations. We identified two fluid flow regimes: CPF and GD. Wettability was found to have an impact on phase‐connectivity, which in turn affects the fluid displacement efficiency. We established that flow transport kinetics of the invading phase are enhanced when the medium deviates away from the neutrally wet state. It was also observed that GD accelerate displacement kinetics. The rate of change of defending phase was the slowest under the neutrally wet condition. For a contact angle of 90°, there was no invading phase break up, mobilization, and GD, and the fluid displacement efficiency was the highest due to the occurrence of lateral fluid invasion. Our findings, however, show that GD are non‐local and cooperative displacement processes whose contribution to fluid flow, fluid configuration, and breakthrough time should not be ignored when conducting continuum scale studies. This will enable us to make more reliable estimates of CO_2_ storage capacity and oil recovery allowing informed and sound business decisions to be made with regards to site selection and development. While in this work 2D simulations have been conducted to establish the effects of wettability on GD, for future work we will consider carrying out 3D simulations to investigate the possible effects of third dimension features such as surface roughness on dynamic fluid connectivity.

## Supporting information

Supporting Information S1

Movie S1

Movie S2

Movie S3

Movie S4

Movie S5

## Data Availability

Data sets and videos supporting the results and discussions are publicly available at Heriot‐Watt University's Research Portal. DOI: 10.17861/5ccc82f4-0b07-4520-87a6-a6d6e5e08977.
