# Supplementary material for: The Impact of Wettability on Dynamic Fluid Connectivity and Flow Transport Kinetics in Porous Media
Source: Water Resour Res. 2022 Jun 3;58(6):e2021WR030729. doi: 10.1029/2021WR030729 (PMC9285789; doi:10.1029/2021WR030729)
Supplement: Supplementary file 1 — Supporting Information S1 [file WRCR-58-0-s003.docx]

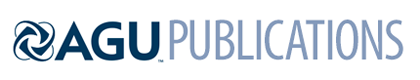


*Water Resources Research*

Supporting Information for

**The Impact of Wettability on Dynamic Fluid Connectivity and Flow Transport Kinetics in Porous Media**

Rumbidzai Nhunduru^1^, Amir Jahanbakhsh^1^, Omid Shahrokhi^1^, Krystian. L. Wlodarczyk^1, 2^, Susana Garcia^1^ and M. Mercedes Maroto-Valer^1^

^1^Research Centre for Carbon Solutions (RCCS), School of Engineering and Physical Sciences, Heriot-Watt University, EH14 4AS, United Kingdom

2Applied Optics and Photonics (AOP) Group, School of Engineering and Physical Sciences, Heriot-Watt University, EH14 4AS, United Kingdom

**Contents of this file**

**Figures S1 to S8**

**Table S1**

**Additional Supporting Information (Files uploaded separately)**

- **Movie S1.** Fluid displacement process and dynamic ganglion behaviour for contact angle 30°
- **Movie S2.** Fluid displacement process and dynamic ganglion behaviour for contact angle 45°
- **Movie S3.** Fluid displacement process and dynamic ganglion behaviour for contact angle 90°
- **Movie S4.** Fluid displacement process and dynamic ganglion behaviour for contact angle 135°
- **Movie S5.** Fluid displacement process and dynamic ganglion behaviour for contact angle 150°

**Introduction**

This supplementary document provides additional information pertaining to mesh refinement in the Berea sandstone microstructure studied as well as data illustrations of quantifications and characterization of residual ganglia in the five contact angle cases investigated i.e.) CA = 30°, 45°, 90°, 135° and 150°. Video files showing fluid displacement time dependent ganglion behaviour for these cases have also been provided


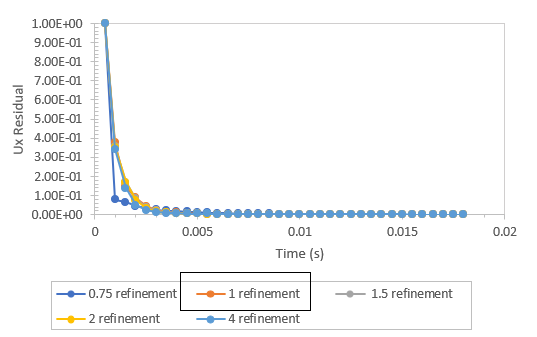


**Figure S1.** Mesh sensitivity study for Berea sandstone model


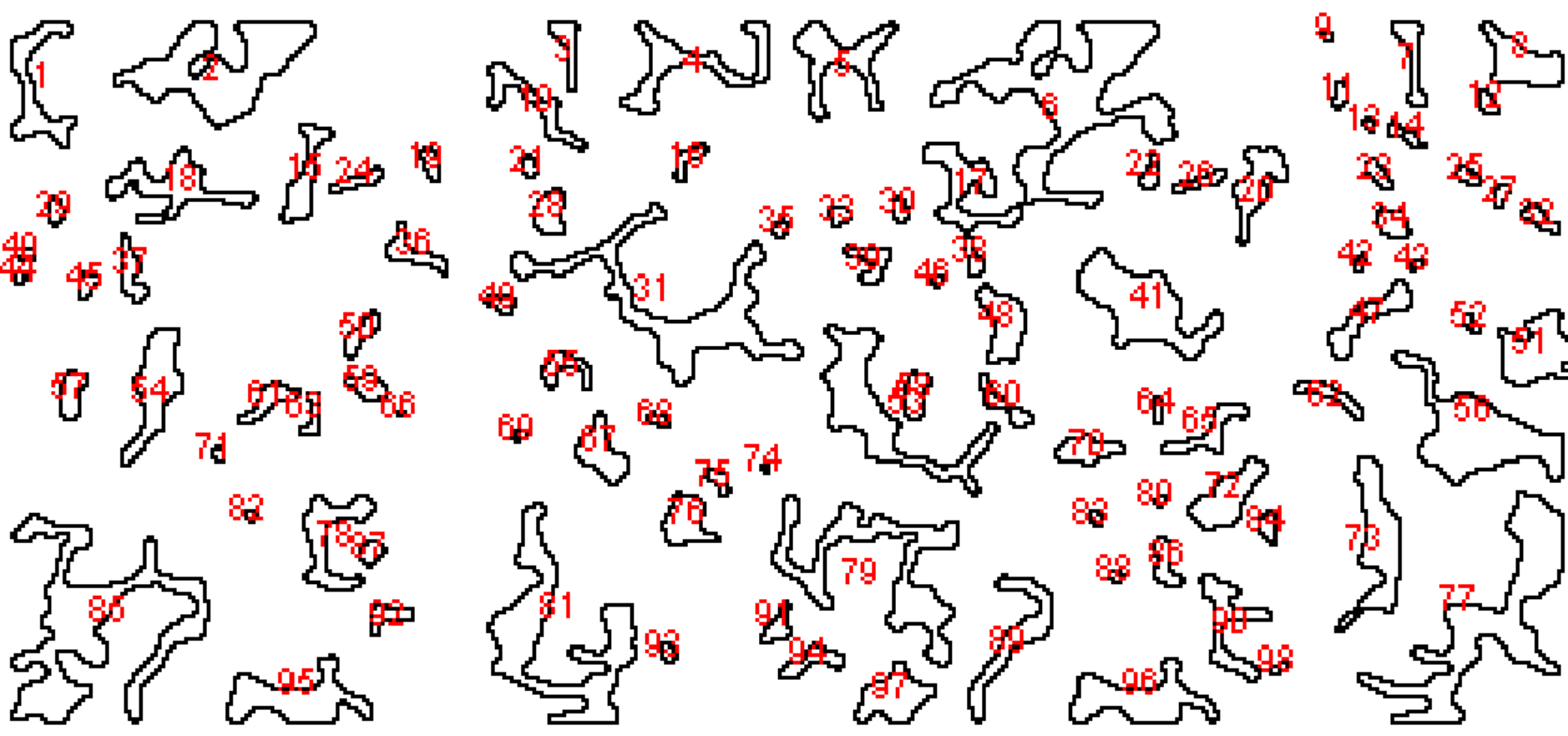


Figure S2. Discretization and Quantification of Ganglia by Image Processing

Table S1. Characterization of Residual Ganglia for 30°, 45°, 90°, 135° and 150° Simulation Cases.

| **Case** | **Number of Ganglia** | **Minimum ganglion size** | **Maximum ganglion size** | **Total Ganglion Area (µm2)** | **Average Ganglion size (µm2)** | **Average Ganglion Perimeter (µm)** |
| --- | --- | --- | --- | --- | --- | --- |
| **30°** | 87 | 23.9 | 29 937.6 | 257 770 | 2 963 | 276 |
| **45°** | 85 | 47.8 | 23 624.9 | 312 050 | 3 671 | 335 |
| **90°** | 65 | 47.8 | 25 513.9 | 203 275 | 3 127 | 272 |
| **135°** | 98 | 47.8 | 24 103.1 | 32 7808 | 3 345 | 311 |
| **150°** | 113 | 23.9 | 23 696 | 33 8879 | 2 999 | 285 |


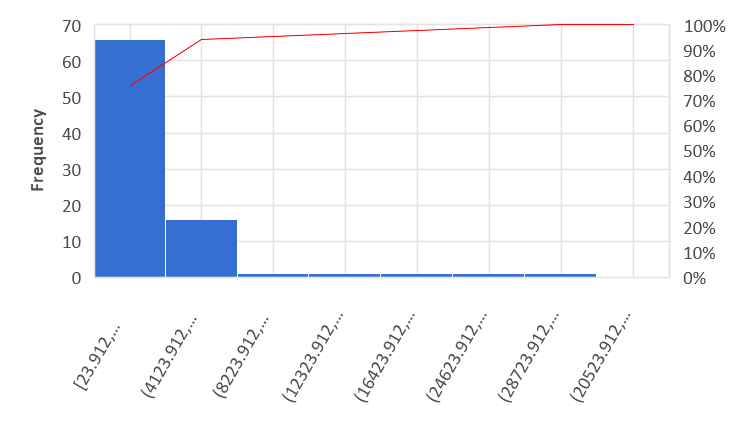


Count: 87 Min: 23.912 µm2

Mean: 2962.872 Max: 29937.650 µm2

Std Dev: 5158.632 Modal Class: 23.912 (66)

Bins: 8 Bin Width: 4100

Figure S3. Residual Ganglion Size Distribution (Area) and Cumulative Frequency for 30° simulation Case


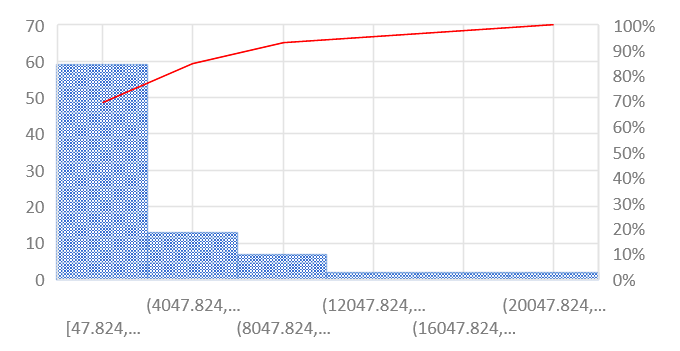


Count: 85 Min: 47.824 µm^2^

Mean: 3671.174 Max: 23624.918 µm^2^

Std Dev: 5086.951 Mode: 47.824 (59)

Bins: 6 Bin Width: 4000

Figure S4. Residual Ganglion Size Distribution (Area) and Cumulative Frequency for 45° simulation Case


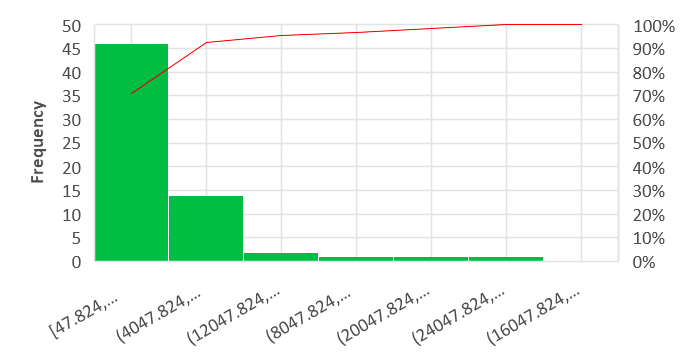


Count:65 Min: 47.824 µm^2^

Mean: 3127.304 Max:25.513.955 µm^2^

Std Dev: 4639.670 Mode: 47.824 (46)

Bins: 7 Bin Width: 4000

Figure S5. Residual Ganglion Size Distribution (Area) and Cumulative Frequency for 90° simulation Case


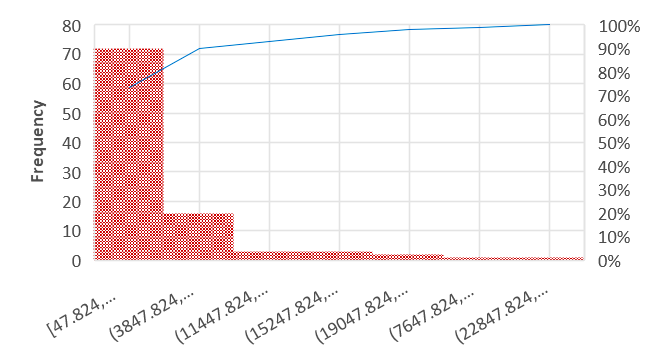


Count:98 Min: 47.824 µm^2^

Mean: 3344.977 Max:23696.654 µm^2^

Std Dev: 5024.414 Mode: 47.824 (73)

Bins: 7 Bin Width: 3800

Figure S6. Residual Ganglion Size Distribution (Area) and Cumulative Frequency for 135° simulation Case


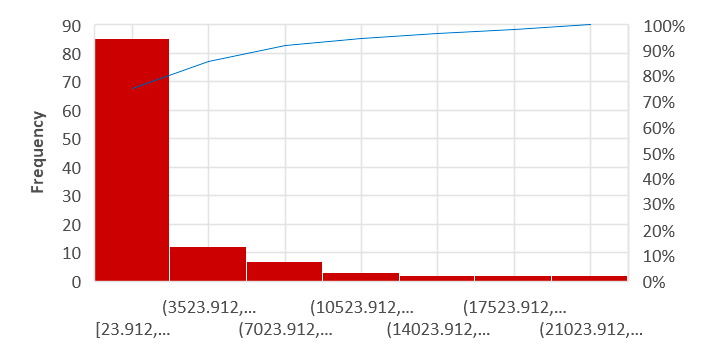


Count:113 Min: 23.912 µm^2^

Mean: 2998.928 Max:23696.654 µm^2^

Std Dev: 4847.847 Mode: 23.912 (84)

Bins: 7 Bin Width: 3500

Figure S7. Residual Ganglion Size Distribution (Area) and Cumulative Frequency for 150° simulation Case


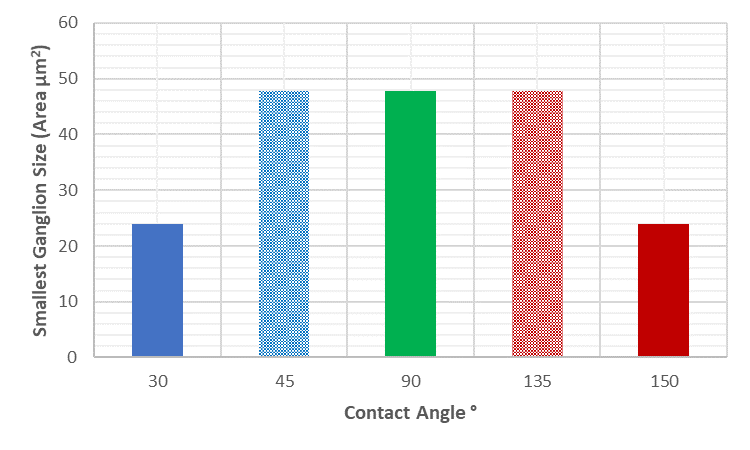


Figure S8. Smallest Residual Ganglion Size (Area) for 30°, 45°, 90°, 135° and 150° Simulation Cases
